# Single center experience with ABO-incompatible and ABO-compatible pediatric heart transplantation

**DOI:** 10.3389/frtra.2024.1452617

**Published:** 2024-10-10

**Authors:** L. Lily Rosenthal, Tabea Katharina Spickermann, Sarah Marie Ulrich, Robert Dalla Pozza, Heinrich Netz, Nikolaus A. Haas, René Schramm, Michael Schmoeckel, Christian Hagl, Jürgen Hörer, Sebastian Michel, Carola Grinninger

**Affiliations:** ^1^ Department of Heart Surgery/Division for Pediatric and Congenital Heart Surgery, Ludwig Maximilian University Munich, Munich, Germany; ^2^ European Pediatric Heart Center Munich (EKHZ), Munich, Germany; ^3^ Department of Congenital and Pediatric Heart Surgery, Technische Universität München, German Heart Center Munich, Munich, Germany; ^4^ Department of Heart Surgery, Ludwig Maximilian University Munich, Munich, Germany; ^5^ Department for Epidemiology and Prevention of Cardiovascular Diseases, Munich Heart Alliance (MHA)—DZHK, Ludwig Maximilian University Munich, Munich, Germany; ^6^ Division of Pediatric Cardiology and Intensive Care, Ludwig Maximilian University Munich, Munich, Germany; ^7^ Clinic for Thoracic- and Cardiovascular Surgery, Heart and Diabetes Center Northrhine Westfalia, Bad Oeynhausen, Germany

**Keywords:** congenital heart failure, ABO-compatible HTx, ABO-incompatible HTx, cardiomyopathy in small children, Norwood failure, congenital heart defect (CHD)

## Abstract

**Introduction:**

The aim of this study was to analyze the results after pediatric heart transplantation (pHTx) at our single center differentiating between ABO-incompatible (ABOi) and -compatible (ABOc) procedures.

**Methods and patients:**

We retrospectively analyzed outcomes of ABO-incompatible HTx procedures performed at our center and compared the data to ABO-compatible HTx of the same era. Eighteen children (<17 months) underwent pediatric HTx and seven of them underwent ABO-incompatible HTx between 2003 and 2015.

**Results:**

Mechanical circulatory support as bridge to transplant was necessary in 3/7 patients before ABO-incompatible HTx and in 3/11 patients before ABO-compatible HTx. Mean waiting time on the list was 36 ± 30 days for ABO-incompatible HTx and 86 ± 65 days for ABO-compatible HTx. The 5-years re-transplant free survival was 86% following ABO-incompatible and 91% after ABO-compatible. In the cohort undergoing ABO-incompatible HTx, 2 patients showed an acute cellular rejection, while early graft failure was not observed. In the cohort undergoing ABOcompatible HTx, acute cellular rejection was observed in 9/11 patients, with early graft failure occurring in nine and CVP in two. A total of ten children were listed for ABO-incompatible HTx after 2015; however, all ten underwent an ABO-compatible transplantation.

**Discussion:**

This study adds much needed information to the literature on ABOi-HTx by showing with a retrospective single center analysis that it is safe and leads to shorter waiting times. We conclude that strategies for ABOi-HTx should be elaborated further, potentially allowing more timely transplantation and thereby preventing waiting list complications such as the need for mechanical circulatory support and even death.

## Introduction

Heart transplant candidates face extended waiting times in Germany due to the persistent shortage of appropriately matching donor organs (1). This notion applies particularly for pediatric patients with advanced heart failure. In fact, small children await heart transplantation markedly longer than adults (2).

Blood group incompatible (ABOi) heart transplantation has been proposed in small children below 24 months of age to potentially shorten waiting times. Physiologically, there is a certain time window of tolerance that exists during human embryonic development and that persists into infancy. Newborns and infants exhibit low levels of isohemagglutinins directed against the blood group determining epitopes and have low levels of T-cell-independent antigens (3–5).

Basically, plasma, erythrocyte concentrates and platelet concentrates can be used for these procedures and transfusions in ABOi HTx, as shown in Supplementary Table S2 in relation to blood group combination. It should also be noted that blood group O is considered a universal donor for erythrocyte transfusions, whereas blood group AB can be used universally for plasma and platelet transfusions (6). Several specialized centers have published their experiences on ABOi-HTx (6–9). Certain threshold levels for isohemagglutinin titers have been proposed underneath which these procedures may be safely performed using specific perioperative immunomodulatory protocols including plasma- or immunpherese, induction therapy and immunoadsorption (10–13).

We herein summarize our single center experiences with ABOi-HTx and compare the results to blood group compatible procedures (ABOc).

## Methods and patients

### Study design

This is an analysis of retrospective data, summarizing our single center experience with ABOi-HTx.

All pediatric patients of less than 24 months of age, who underwent ABOi-HTx were identified. Clinical data on pre-, peri- and postoperative conditions were retrieved from the prospectively run clinical data base and archived patient files. To better contextualize the data, we also analyzed all ABOc-HTx procedures performed during the same era.

### Immunosuppression

Induction therapy was used in all patients. We used either basiliximab (Simulect®; Novartis Pharma GmbH, Basel, Switzerland) (*n* = 1). or anti-thymocyte globulin (ATG; Atgam®, Pfizer Pharma GmbH, Berlin, Germany) (*n* = 6). There were no defined criteria which induction therapy was used. All patients undergoing ABOi HTx received plasma exchange on cardiopulmonary bypass during transplant surgery until isohemagglutinin were no more detectable (Supplementary Table S2). Intravenous methylprednisolone (30 mg/kg) and ATG was given before release of the aortic cross-clamp.

In all patients, maintenance triple immunosuppression was based on a calcineurin inhibitor (cyclosporin A or tacrolimus), the anti-metabolite mycophenolate mofetil (MMF) and a glycocorticoid (Supplementary Table S5) in accordance with international standard protocols and adjusted to the patients’ individual conditions and needs (14, 15).

All patients with mismatch of CMV IgG received CMV hyperimmunoglobulin once (Cytotect R®, Cytoglobin R®) and Valganciclovir for 3 months post transplant. Patients received P. carinii pneumonia prophylaxis for 1 year and antifungal prophylaxis with oral amphotericin B (Ampho moronal®) over a period of 3–6 months.

Acute cellular rejections (ACR) were categorized according to the pathological findings in endomyocardial biopsies and graded according to the ISHLT consensus (16, 17). Treatment of ACR was indicated at ISHLT grade ≥2 by pulsed glucocorticoids.

### Statistics

Data were analyzed using SPSS 29 statistics software (IBM®, Armonk, Armonk, New York, USA). For descriptive analyses, continuous variables were expressed as mean values ± standard deviation (range) and categorical variables were expressed as frequencies. Comparisons between parameters in ABOi- and ABOc-HTx, we used Man Whitney *U*-test for not normally distributed data for continuous variables and Qui Square Test for categorical variables. Survival data were analyzed using the Kaplan Meier method. Differences between groups were described.

## Results

A part of our results was already presented in prior studies of our center (8, 9, 18–20). From 2003 to 2023, a total of 31 patients, all of whom were less than 24 months of age, had been listed for HTx, 7 of them were transplanted by ABOi between 2003 and 2015 (Supplementary Table S1). Two patients presented with congenital heart disease in addition to hypoplastic left heart syndrome and had not undergone a prior palliation operation. In the same period, 11 ABOc HTx procedures were conducted and constituted as control group. Of these, six were observed to have congenital heart disease. Four of the six patients had hypoplastic left heart syndrome, with two cases presenting with Norwood failure and four with Glenn (partial cavo-pulmonary connection) failure. Two patients in the ABOi HTx group and three patients in the ABOc HTx group required LVAD (left ventricular assist device) support, as shown in Supplementary Table S3. The antibody titer of recipients from the ABOi HTx group was available at the time of transplantation (Supplementary Table S4). All patients in the ABOi HTx group underwent intraoperative plasma exchange on cardiopulmonary bypass during the transplantation process until isohemagglutinin was no longer detectable. The individual antibody titers against blood group antigens A1, A2, and B, plotted against the ratio of donor to recipient blood groups are presented in Supplementary Table S4. It is evident that no patient in this cohort observed an antibody titer exceeding 1:8 spontaneous. Two patients showed no distinct antibody titers, two patients demonstrated titers for all three examined characteristics, and the remaining patients showed a titer of 1:4 against one or both subgroups of anti-A antibodies.

The preoperative patient characteristics are depicted in Supplementary Table S3. Briefly, the mean recipient age at the time of transplant was 1 ± 15 months, i.e., 7 ± 4 months in ABOi and 6 ± 4 months in ABOc-HTx. Donors were 9 ± 7.5 months of age, i.e., 10 ± 4 months in ABOi and 9 ± 9 in ABOc-HTx. The mean waiting time was 66.5 ± 58.5 days, i.e., 36 ± 30 days in ABOi and 86 ± 65 days in ABOc-HTx. The last isohemagglutinin titers before transplant and the different donor/recipient blood group combinations in ABOi-HTx are depicted in Supplementary Table S3. There were 8 children supported by mechanical circulatory support (Supplementary Table S3), 4 in ABOi and 4 in ABOc-HTx. There were 4 transplants using blood group 0 donor hearts in recipients with blood group A (*n* = 2), B (*n* = 1), and AB (*n* = 1), self-evidently following standard procedures for ABOc-HTx.

Intraoperative data are depicted in Supplementary Table S6. In brief, cardiopulmonary bypass (CPB) times were 288 ± 72 min, i.e., 272 ± 87 min in ABOi and 298 ± 63 in ABOc-HTx. Overall donor organ ischemic times were 221 ± 57 min, i.e., 216 ± 80 min in ABOi and 221 ± 60 min in ABOc-HTx. Warm ischemic times were 116 ± 61 min, i.e., 99 ± 64 min in ABOi and 127 ± 60 min in ABOc-HTx. Four patients were supported by extracorporeal membrane oxygenation directly after transplant, three of them for 2 days in ABOi and one for 2 days in ABOc-HTx (Supplementary Table S6).

Post-transplant data are depicted in Supplementary Table S7, S8. Briefly, mean times on postoperative invasive mechanical ventilation were 168 ± 120 h, i.e., 216 ± 120 h min in ABOi and 120 ± 96 h in ABOc-HTx. Lengths of hospital stays were 148 ± 103 days, i.e., 122 ± 69 days in ABOi and 165 ± 120 days in ABOc-HTx. Lengths of post-HTx hospital stays were 84 ± 68 days, i.e., 56 ± 17 days in ABOi and 73 ± 68 days in ABOc-HTx.

The mean follow-up times were 12 ± 6 years, i.e., 11 ± 3 years in ABOi and 13 ± 2 years in ABOc-HTx. Overall survival after transplant was 61% (0.5–21 years)., i.e., 1–19 years in ABOi and 0,5–20 years in ABOc-HTx. Survival after ABOi-HTx was 100%, 86% and 69% at 1, 5 and 10 years, respectively. Four patients died following ABOi-HTx. One patient required re-transplant because of severe hypertrophic cardiomyopathy. Survival after ABOc-HTx was at 1, 5 and 10 years 100%, 91% and 82%, respectively. Three patients died following ABOc-HTx. Causes of deaths included malignancies, acute and chronic graft failure. None of the patient required re-transplantation after ABOc-HTx (Supplementary Table S8).

Data on rejection episodes are depicted in Supplementary Table S7. Relevant acute cellular rejections (ACR; ISHLT grade >= 2R) occurred in one patient, i.e., one in ABOi- and in none of patients after ABOc-HTx.

CAP (Coronary artery vasculopathy) observed after 2 years, i.e., 1.5 ± 1 years in ABOi and 2 ± 1 years after ABOc-HTx.

Relevant infections in the first year after transplant occurred in 5 patients, i.e., 1 patients in ABOi and 4 in ABOc-HTx. Post-transplant malignancies developed in 8 patients, i.e., 3 patients in ABOi and 5 in ABOc-HTx (Supplementary Table S8). Delayed chest closure has been performed in three patients each group for persistent diffuse bleeding and/or myocardial edema. Redo-sternotomy because of bleeding or effusion was not necessary.

### Characteristics of the ABOi HTx patients

Patient 1 (female) developed severe subaortic stenosis subsequent to ABOi-HTx (21), which was subsequently repaired via two surgical repairs. Subsequently, approximately six years later, she underwent ABOc-HTx due to the development of severe hypertrophic cardiomyopathy.

Patient 2 (male) is currently alive and in good health.

Patient 3 (female) died 14 years following her transplantation because of urethra carcinoma and urinary bladder carcinoma with lymphatic metastasis.

Patient 4 (male) died after 10 years post-transplant as a consequence of severe humoral rejection and cardiogenic shock.

Patient 5 (male) died nine years following his transplantation as a result of neuroendocrine carcinoma with high malignancy

Patient 6 (female) died one year following her transplantation due to a fulminant, therapy-refractory bacterial and fungal infection with severe renal and multiorgan failure.

Patient 7 (male) developed chronic rejection due to medication incompliance of the parents soon after transplantation. This was the only patient who needed biVAD support eight years after his initially ABO incompatible HTx. He is listed for re-transplantation. This was the only patient who developed donor-specific antibodies and therefore was treated with rituximab for several times. Two of them presented antibody titers, but did not exhibit any signs of rejection during the subsequent clinical course following ABOi HTx. The available HLA-typing data of donor and recipient as well as the HLA-A-B-DR mismatches is shown in Supplementary Table S9.

### Causes of death after ABOc-HTx

Patient 10 (female) died 10 years following her transplantation procedure due to renal cell carcinoma with peritoneal carcinosis.

Patient 15 (female) died 18 days post-transplant because of severe hypoxic brain injury resulting and severe therapy-refractory early graft failure.

Patient 17 (female) died eight years after her transplantation due to fulminant, therapy-refractory rejection with acute graft failure.

## Discussion

This analysis shows our single-center experience with ABOi-HTx and compares pre- peri- and postoperative data with ABOc-HTx of the same era. Our data underline previous experiences showing that ABOi-HTx is safe and reaches comparable outcomes to ABOc procedures (8, 9, 18–22). The possibility for ABOi-HTx may expand the donor organ pool for an individual recipient and accelerate the allocation process. Eventually, donor organs could be distributed more effectively and cold ischemic times could potentially be shortened. One important potential advantage is, that it may help to use organs which might be discarded for blood group incompatibility purposes. However, despite the latter, more vague advantages and the potentially expanded donor pool in the individual case, ABOi-HTx will most likely not allow a significant increase in overall transplant numbers, given the restricted global number of organ donors.

Nevertheless, we and others (7, 21) have found reduced waiting times in patients undergoing ABOi-HTx and this is obviously a paramount feature in the individual patient development. Scale investigations will have to clarify whether the uneven distribution of blood groups in every population impact on such findings. Urschel et al. reported that patients with blood type 0 had a shorter mean time on the waiting list for a suitable heart (23). Given the restricted donor heart source, it is of course beneficial to reduce waiting times *per se*, but it needs to be evaluated whether ABOi-HTx may actually reduce overall wait list mortality, which is probably the most important parameter in times of donor organ shortage. Almond et al. demonstrated in their results no significant impact on waiting list on mortality, but they found that candidates for ABOi-HTx were more ill and required more assist device support (7, 21). West at al. reported that listing patients for ABO_i_ HTx reduced the waiting list mortality (24). Such data are difficult to obtain, as only some centers perform ABOi-HTx and it is difficult to distinguish which patient with a fatal course on a ABOc-HTx waiting list could have effectively saved by ABOi HTx. In line with this, there is no standardized protocol for listing for ABOi-HTx. We can only speculate whether we could have transplanted more patients earlier if we would have accepted higher threshold levels of isohemagglutinin titers. In fact, Urschel et al. demonstrated safe ABOi-HTx in individuals with isohemagglutinin titers beyond our limits (12), which could be used in older age and with higher isohemagglutinin titers than initially assumed and using similar immunosuppressive regimens as for ABO-compatible transplants (25). At our center a maximum titer of 1:8 was measured before ABOi HTx. This occurred only once and, like all other values, were reduced by the pre- and perioperative procedures already described. International studies report in individual cases, especially in older children, significantly higher titer values at initial listing (1:256), which, however, were also reduced to a value of ≤1:16 during the preoperative course (26). With increasing experience in the field of incompatible transplantation modalities, listing with pre-existing antibodies are becoming more common. This goal has been achieved with the available measures for titer reduction and has been confirmed as a safe procedure by the experience gained. The fact that AB0i HTx has already been successfully transplanted at our institute despite pre-existing antibodies is a gratifying result and promising for further expansion. In principle, it is possible to list patients with initially higher antibody titers for AB0i HTx as shown by Krauss et al. (25). With regard to the question of an increased risk of rejection in the postoperative course, the so-called donor-specific antibodies (DSA) must be also taken into account. The development of DSA is a significant risk factor for the development of antibody-mediated graft rejection. The development of DSA is generally observed in 10%–30% of patients after HTx. DSA is also associated with decreased donor and patient survival. Adherence to consistent and sufficient immunosuppression appears to be essential to minimize the risk of new DSA and thus rejection, and induction therapy with ATG has also shown promising results (27). However, international data show that patients after ABOi HTx are less likely to develop specific antibodies. Thus, the initially feared risk of rejection associated with antibody formation is lower in this patient group than in patients after ABOc HTx (28). Individual case reports, such as that of a patient who developed multiple humoral rejections in the postoperative course despite the absence of risk factors for a rejection, illustrate the importance of active monitoring of antibody formation and the most precise control of all controllable parameters, as these limit the survival of transplant patients (29). The growing experience with successful heart transplantations across positive HLA crossmatches by use of distinct desensitization protocols may help to promote ABOi HTx. In our study two patients developed donor-specific antibodies, one of them due to low immunosuppressive therapy given by the parents. The other patient died due to acute humoral rejection 11 years post-transplant. Foreman et al. described in 2002 that in the 16 children who underwent AB0i transplantation, preoperative blood group, i.e., ABO system, titers were determined and repeated after successful HTx. In these transplants, blood plasma was exchanged during the intraoperative period of cardiopulmonary bypass to eliminate existing antibodies against the blood group antigens. The heart-lung machine required during HTx was loaded with an additional volume of recipient compatible red cells, plasma, and platelets free of all anti-A and anti-B antibodies to replace the patient's blood. The volume of blood removed from the recipient by the heart-lung machine was also subjected to remove any remaining antibodies with the plasma, and only the red blood cells were finally returned to the bloodstream (13). In our center we used the same technique for plasma exchange transfusion (Supplementary Table S2). As an extension of this procedure, 30 successful ABOi HTx were reported in the United Kingdom in 2015 in patients with preoperative antibody titers ≥1:16 who received a so-called plasma exchange transfusion (PET) in the pre- and perioperative period (30, 31). Robertson et al. reported in 2018 that preoperative isohemagglutinin titers were reduced by an anti-A/anti-B immune absorption in the extracorporeal limb of the heart-lung machine. In addition to conserving blood products, patients with higher preoperative antibody titers may be candidates for AB0i HTx. In addition, the use of immune absorbers, also in combination with PET, offers the possibility of offering larger and therefore older patients a blood group incompatible listing and transplantation (32). Clinical aspects such as the use of ventricular assist devices, which are associated with possible sensitization to blood group antigens, and the possible development of B-cell tolerance in early childhood after ABOi HTx must also be considered (22, 33, 34). Further, novel desensitization protocols for heart transplantation in immunized patients with donor-specific HLA antibodies (26) may be adopted potentially elaborating on current strategies in ABOi HTx. These questions require further investigations and multi-center studies. These questions require further investigations and multi-center studies.

Analyzing survival is probably the most objective means to evaluate safety of ABOi-HTx. In fact, data from the United Network for Organ Sharing (UNOS) may be considered to represent « a critical mass of evidence » adjusting for existing significant risk factors (7, 21, 35–37). Everitt analyzed a multi-center population of 1029 patients listed for HTx consisting of 277 patients listed for ABOi-HTx (27%) and 752 listed for ABOc-HTx (73%). During the study period, the number of patients listed for ABOi-HTx increased from 5.8% (9 of 154) in 2001 to 43.9% (65 of 148) in 2007, potentially the result of increased experience with ABOi-HTx (38). In our population from 2003 to 2023, 31 patients (<24 months) had been listed for pHTx, 5 for a compatible and 26 for incompatible heart transplantation. A total of 7 ABOi-HTx were performed at our center between 2003 and 2015. 7 patients had been never received any matching donor heart and were transplanted with an ABOc organ above 24 months of their age. (Supplementary Table S1).

However, analyzing post-transplant morbidity is equally important. It needs to be addressed, whether the distinct immunomodulatory protocol for ABOi-HTx may be associated with enhanced episodes of infection or incidences of malignancies, or if the use of a blood group incompatible donor organ may accelerate rejections. Henderson et al. reported that 75% of the patients who underwent ABOi-HTx remained free from post-HTx rejection, compared with only 63% of the patients who received ABOc organs. The infection rate was also lower in the ABOi-HTx group (23.5% vs. 37.9%) (39). Roche et al. demonstrated that development of hypertension after pHTx, as a common problem, is associated with immunosuppressive regimes (40, 41). In 2001, West et al. demonstrated an 80% survival rate in 10 patients after ABOi-HTx. In their experience, death was not the result of hyperacute rejection triggered by Donor-specific antibodies (6, 28, 33, 42). A multicenter analysis by UNOS showed that the incidence of PTLD 1 year post HTx was 14% after ABOc-HTx and 13% after ABOi-HTx. There was no significant difference between the groups (43, 44). The development of PTLD was associated with the presence of EBV infection (45, 46) due to the dysregulation of the immune system caused by the viral infection. A current focus of research involves identifying specific biomarkers and developing therapeutic approaches. Promising approaches that consider a combination of analyzed molecules and viral load need to be further investigated and evaluated, especially in the pediatric patient population (46).

## Limitations

Of our investigation is the precisely defined and available clinical data in a single patient cohort on ABOi HTx, which is rare. There is a lot of interest in this specific data, but the limited number of patients don't allow conclusions on a broader scale. However, they may add to the collective data available from other centers and thereby improve outcomes in these patients.

## Conclusion

This study adds much needed information to the literature on ABOi-HTx by showing with a retrospective single center analysis that it is safe and leads to shorter waiting times. We conclude that strategies for ABOi-HTx should be elaborated further, potentially allowing more timely transplantation and thereby preventing waiting list complications such as the need for mechanical circulatory support and even death.

## Data Availability

The datasets presented in this study can be found in online repositories. The names of the repository/repositories and accession number(s) can be found in the article/Supplementary Material.
