# Supplementary material for: Single center experience with ABO-incompatible and ABO-compatible pediatric heart transplantation
Source: Front Transplant. 2024 Oct 10;3:1452617. doi: 10.3389/frtra.2024.1452617 (PMC11499225; doi:10.3389/frtra.2024.1452617)
Supplement: Supplementary file 1 [file Datasheet1.docx]

Supplementary Material

# Supplementary Data

# Supplementary Tables

**Table 1: The Number of pediatric heart transplantations in Germany from 2003 – 2023 in patients <16 years (Euro transplant international foundation’s data) comparing with data from our center. Listing for HTx in patients ≤ 2y and count of HTx in patients ≤ 2 years at our center.**

| **Transplant year** | **Blood group compatibility** | **Number of HTx**  **in Germany** | **All HTx**  **in Germany** | **All HTx at our center** | **Listing for HTx**  **in at center** | **Number of HTx ≤ 2y at our center (blood group compatibility)** | **All HTx in patients ≤ 2y at our center** |
| --- | --- | --- | --- | --- | --- | --- | --- |
| **2003 - 2007** | Compatible | **134** | 139 | 32 | 3 | 5 | 10 |
| **2003 - 2007** | Incompatible | **5** |  |  | 7 | 5 |  |
| **2008 - 2012** | Compatible | **147** | 149 | 22 | 2 | 7 | 8 |
| **2008 - 2012** | Incompatible | **2** |  |  | 6 | 1 |  |
| **2013 - 2017** | Compatible | **186** | 194 | 20 | 0 | 2 | 3 |
| **2013 - 2017** | Incompatible | **8** |  |  | 8 | 1 |  |
| **2018 - 2023** | Compatible | **235** | 248 | 16 | 0 | 3 | 3 |
| **2018 - 2023** | Incompatible | **13** |  |  | 5 | 0 |  |
| **2003-2023** | All | **730** |  | 90 | 31* | 24 | 24* |

**Abbreviations and Acronyms:** HTx: Pediatric heart transplantation. Descriptive analysis: categorical variables were expressed as frequencies with count; SPSS^®^. * 7/31 patients were transplanted above 24 months of age.

**Table 2: Blood group donor / Blood group recipient: Plasma exchange according to West et al*.**

| **Donor’s blood group** | **Recipient’s blood group** | **Antibodies to avoid** | **Indicated blood group** | | |
| --- | --- | --- | --- | --- | --- |
|  |  |  | **Plasma** | **Red cells** | **Platelets** |
| **AB** | O | Anti-A (vs. graft)  Anti-B (vs. graft) | AB | O | AB |
| **B** | O | Anti-B (vs. graft) | AB or B | O | AB or B |
| **A** | O | Anti-A (vs. graft) | AB or A | O | AB or A |
| **AB** | B | Anti-A (vs. graft)  Anti-B (vs. graft and recipient) | AB | O or B | AB |
| **A** | B | Anti-A (vs. graft)  Anti-B (vs. recipient) | AB | O or B | AB |
| **AB** | A | Anti-B (vs. graft)  Anti-A (vs. graft and recipient) | AB | O or A | AB |
| **B** | A | Anti-B (vs. graft)  Anti-A (vs. recipient) | Ab | O or A | AB |

*****West LJ. ABO-incompatible hearts for infant transplantation. Current opinion in organ transplantation. 2011;16(5):548-54.

**Table 3: Characteristics of recipients with ABO-incompatible or ABO-compatible Donors at time to transplantation (n).**

|  | **Age (mo)** | **Weight (kg)** | **BMI**  **(kg/cm^2^)** | **Diagnosis** | **Recipient’s Blood group** | | **Donor’s Blood group** | **Pre-HTx ECLS/VAD** | **Waiting time on the list (d)** |
| --- | --- | --- | --- | --- | --- | --- | --- | --- | --- |
| **Patient 1** | 7 | 12 | 4 | HLHS | | O | A | ECLS | 78 |
| **Patient 2** | 5 | 12 | 3 | HLHS | | O | A | - | 20 |
| **Patient 3** | 3 | 18 | 7 | DCM | | O | B | - | 2 |
| **Patient4** | 6 | 14 | 5 | DCM | | O | AB | ECLS | 8 |
| **Patient 5** | 16 | 16 | 4 | DCM | | O | B | - | 33 |
| **Patient 6** | 10 | 11 | 5 | DCM | | O | B | ECLS/LVAD | 35 |
| **Patient 7** | 7 | 13 | 5 | DCM | | A | B | ECLS/LVAD | 73 |
| **Patient 8** | 5 | 14 | 5 | HLHS | | O | O | - | 6 |
| **Patient 9** | 1 | 12 | 3 | DCM | | A | A | ECLS | 10 |
| **Patient 10** | 3 | 15 | 6 | HLHS | | B | O | - | 68 |
| **Patient 11** | 3 | 14 | 7 | HLHS | | AB | O | - | 68 |
| **Patient 12** | 8 | 15 | 7 | HLHS | | O | O | - | 85 |
| **Patient 13** | 6 | 10 | 4 | DCM | | O | O | - | 69 |
| **Patient 14** | 2 | 13 | 5 | DCM | | O | O | - | 226 |
| **Patient 15** | 15 | 11 | 3 | ALCAPA | | A | O | - | 45 |
| **Patient 16** | 9 | 15 | 8 | Myocarditis | | O | O | LVAD | 78 |
| **Patient 17** | 12 | 13 | 9 | DCM | | A | O | LVAD | 137 |
| **Patient 18** | 8 | 15 | 6 | DCM | | A | A | LVAD | 156 |

**Abbreviations and Acronyms:** ABO: Blood group. BMI: Body mass index. HTx: Heart transplantation. ECLS: Extra Cardiac Life Support. VAD: Ventricular assist device. HLHS: Hypoplastic left heart syndrome. DCM: Dilatative cardiomyopathy. ALCAPA: Anomalous left coronary artery from pulmonary artery (Bland-White-Garland-Syndrome). LVAD: Left ventricular assist device. Descriptive analysis: categorical variables were expressed as frequencies with count; SPSS^®^.

**Table 4: Antibody titer in ABOi-HTx at time of HTx (Donor / Recipient).**

|  | **Donor / Recipient** | **Anti-A1 titer** | **Anti-A2 titer** | **Anti-B titer** | **Rejection*** |
| --- | --- | --- | --- | --- | --- |
| **Patient 1** | **A+/O+** | 1:4 | 1:4 | 0 | yes |
| **Patient 2** | **A+/O-** | 1:4 | 0 | 0 | no |
| **Patient 3** | **B+/O+** | 0 | 0 | 0 | no |
| **Patient 4** | **AB+/O+** | 1:4 | 1:4 | 1:1 | yes |
| **Patient 5** | **B+/O-** | 1:8 | 1:2 | 1:2 | yes |
| **Patient 6** | **B+/O+** | 1:2 | 1:0 | 1:1 | no |
| **Patient 7** | **A+ /B+** | 0 | 0 | 0 | yes |

**Abbreviations and Acronyms:** ABOi: ABO incompatible heart transplantation. (A, B, AB, O): Blood group. Descriptive analysis: categorical variables were expressed as frequencies with count; SPSS^®^.

**Table 5: Post- transplant immunosuppressive regime.**

|  | **ABOi-HTx (n)** | **ABOc-HTx (n)** | **All (n)** |
| --- | --- | --- | --- |
| **Initial therapy**   - **CyA, MMF** - **Tac, MMF** - **CyA, Everolimus** | 0  7  0 | 3  7  1 | 3  14  1 |
| **Therapy at time to follow up**   - **CyA, Aza** - **CyA, MMF** - **Tac, Aza** - **Tac, MMF** - **FK 500 as mono therapy** - **Tac, Everolimus** | 1  0  2  0  0  2 | 0  1  2  1  1  4 | 1  1  4  1  1  6 |
| **Reason of changes**   - **Renal failure** - **Intolerance** - **Missing of data** | 2  1  2 | 0  5  4 | 2  6  6 |

Abbreviations and Acronyms: HTx: Pediatric heart transplantation. CyA (CsA): Cyclosporine A: Calcineurin inhibitor. Aza: Azathioprine. MMF: mycophenolate-mofetil. Tac: Tacrolimus (Prograf^®^). Descriptive analysis: categorical variables were expressed as frequencies with count; SPSS^®^.

**Table 6: Patients basic hospital data.**

| **Perioperative data of recipients** | **ABOi-HTx** | **ABOc-HTx** | **All** |
| --- | --- | --- | --- |
| **Waiting time on the list (days)** | 36±30 (2-78) | 86±65 (6-226) | 66.5±58.5 (2-226) |
| **Aortic cross-clamp time (min.)** | 62±8 (49-75) | 95±33 (39-158) | 82±31 (39-158) |
| **Cold ischemic time (minutes)** | 216±80 (123-301) | 221±60 (83-285) | 221±57 (83-301) |
| **Length of VS (hours)** | 216±120 (24-672) | 120±96 (24-120) | 168±120 (24-624) |
| **ICU-Stay (days)** | 18±8 (6-28) | 17 ±8 (9-31) | 18±8 (6-31 |
| **Hospital-length (days)** | 122±69 (57-263) | 165±120 (43-476) | 148±103(43-476) |
| **Hospital-Stay after HTx (days)** | 56±17 (37-84) | 73±68 (18-396) | 84±68 (18-396) |

**Abbreviations and Acronyms:** VS: Ventilation support. ICU: Intensive care unit. Descriptive analysis: Continuous variables were expressed as mean values ± standard deviation (minimum - maximum) and categorical variables were expressed as frequencies with count; SPSS^®^.

**Table 7: Post- transplant results - comparing ABOc HTx to ABOi HTx.**

|  | **ABO_i_ (n)** | **ABO_c_ (n)** | **All (n)** |
| --- | --- | --- | --- |
| **Post HTx ECLS** | 3 | 1 | 4 |
| **Post HTx rejection**   - **Grade 1R (mild)** - **Grade 2R (moderate)** - **Grade 3R (severe)** - **Chronic humoral** | 0  1  1  1 | 0  0  2  0 | 0  1  3  1 |
| **Post HTx early graft failure** | 0 | 1 | 1 |
| **Post HTx late graft failure (WHO)**  **Grade I**  **Grade II**  **Grade III**  **Severe (Grade 4)** | 0  0  0  1 | 7  1  1  0 | 7  1  1  1 |
| **Post HTx EBV infection** | 1 | 7 | 8 |
| **Post HTx CAP**  **CAP I**  **CAP II**  **CAP III** | 1  0  1 | 1  1  0 | 2  1  1 |
| **Post HTx CMV infection** | 1 | 0 | 1 |
| **Post HTx PTLD** | 2 | 5 | 7 |
| **Post HTx tumor**   - **Ureteric carcinoma*** - **Neuroendocrine carcinoma** - **Renal cell carcinoma** - **Bladder cancer*** | 1*  1  0  1* | 0  0  1  0 | 1*  1  1  1* |
| **Post HTx renal failure** | 4 | 8 | 12 |
| **Post HTx dialysis** | 1 | 1 | 2 |
| **Post HTx hypertension** | 4 | 7 | 11 |

**Abbreviations and Acronyms:** ABOi: ABO incompatible heart transplantation (HTx). (A, B, AB, O): Blood group. ABOc: ABO compatible HTx. ECLS: Extra Cardiac Life Support. (0R, 1R, 2R, 3R): Stanford classification: Grad of rejection. EBV: Ebstein-Barr-virus. CMV: Cytomegalovirus. CAP: Stanford classification: Coronary artery vasculopathy. *Ureteric carcinoma combined with bladder cancer. Descriptive analysis: categorical variables were expressed as frequencies with count; SPSS^®^.

# Table 8: Overview of post HTx results in the particular patient

|  | **Status at follow-up-date** | **rejection** | **Graft failure** | **CAP** | **Post-HTx-CMV** | **PTLD/ tumor** | **Post-HTx-EBV** |
| --- | --- | --- | --- | --- | --- | --- | --- |
| **ABOi-HTx** | | | | | | | |
| **Patient 1** | Re-HTx | - | - | - | - | - | - |
| **Patient 2** | Alive | 2R | No | No | No | No | Yes |
| **Patient 3** | Dead | 0R | No | CAP I | No | Urological malignancy (high grad) | No |
| **Patient 4** | Dead | 3R | No | No | No | No | No |
| **Patient 5** | Dead | 0R | No | No | Yes | Yes (neuro endocrine ca.) | No |
| **Patient 6** | Dead | 0R | No | No | No | Yes | No |
| **Patient 7** | Alive | Chronic | WHO III | CAP II-III | No | No | No |
| **ABOc-HTx** | | | | | | | |
| **Patient 8** | Alive | 0R | WHO I | No | Yes | No | Yes |
| **Patient 9** | Alive | 0R | WHO I | No | No | Yes – stadium IV | Yes |
| **Patient 10** | Dead | 0R | WHO I | No | Yes | Yes – B-cell- lymphoma | Yes |
| **Patient 11** | Alive | 0R | WHO I | CAP I | No | Yes (kidney) | No |
| **Patient 12** | Alive | 0R | WHO I | No | No | Yes (intestinal) | Yes |
| **Patient 13** | Alive | 0R | WHO I | No | No | No | No |
| **Patient 14** | Alive | 0R | WHO I | CAP II | No | No | Yes |
| **Patient 15** | Dead | 3R | WHO III | No | No | No | No |
| **Patient 16** | Alive | 0R | No | No | No | No | No |
| **Patient 17** | Dead | 3R | WHO II | No | No | Yes (polymorph) | Yes |
| **Patient 18** | Alive | 0R | No | No | No | Yes (b cell lymphoma) | Yes |

**Abbreviations and Acronyms:** ABOi: ABO incompatible heart transplantation (HTx). (A, B, AB, O): Blood group. ABOc: ABO compatible HTx. CAP: Coronary artery vasculopathy (Stanford classification). Ca: Carcinoma. 0R, 1R, 2R and 3R: Grad of rejection (Stanford classification).

**Table 9: HLA-typing data of donor and recipient as well as the HLA-A-B-DR mismatches.**

| **HLA titer** | | | **HLA-A-B-DR mismatch** |
| --- | --- | --- | --- |
| **Patient 1** | **Donor** | A2 A3 B15 B62 B35 BW6 Cw3 Cw4 DR2 DR16 DR6 DR13 DR51 DR52 DQ1 DQ5 DQ6 | 2-2-1 |
|  | **Recipient** | A9 A23 A19 A30 B16 B38 B13 Cw6 Cw12 DR6 DR13 DR7 DQ2 DQ6 |  |
| **Patient 2** | **Donor** | A3 A19 A29 B7 B12 B44 Bw4 Bw6 DR2 DR15 DR7 DR51 DR53 | 1-1-2 |
|  | **Recipient** | A19 A32 B12 B44 B40 B61 DR5 DR11 DR6 DR13 DQ7 DQ6 |  |
| **Patient 3** | **Donor** | A2 A3 B12 B44 B35 Bw4 Bw6 Cw4 Cw7 DR1 DR5 DR11 DR52 | 0-2-1 |
|  | **Recipient** | A2 A3 B17 B57 B7 DR3 DR7 DQ2 DQ9 |  |
| **Patient 4** | **Donor** | A2 A29 A19 B12 B44 DR7 DR15 DR2 | 1-2-1 |
|  | **Recipient** | A2 A3 B17 B57 B22 B55 DR6 DR14 DR7 DR52 DR53 DQ1 DQ5 DQ9 |  |
| **Patient 5** | **Donor** | A1 A11 B8 B51 Bw4 Bw6 Cw6 Cw7 DR3 DR11 DR5 DQ2 DQ3 | 2-2-2 |
|  | **Recipient** | A2 A3 B17 B57 B22 B55 DR6 DR14 DR7 DR52 DR53 DQ1 DQ5 DQ3 |  |
| **Patient 6** | **Donor** | ***** | **-** |
|  | **Recipient** | A2 A3 B21 B50 B27 Cw2 Cw6 DR2 DR15 DR7 DQ1 DQ6 DQ2 |  |
| **Patient 7** | **Donor** | A3 A19 A31 B12 B44 B16 B39 Bw4 Bw6 Cw12 Cw16 DR6 DR14 DR7 DR52 DR53 DQ1 DQ5 DQ2 | 2-2-1 |
|  | **Recipient** | A19 A29 A2 B27 B41 Cw2 Cw17 DR2 DR16 DR7 DQ1 DQ5 DQ2 |  |

***** Not available (donor outside Eurotransplant)

**
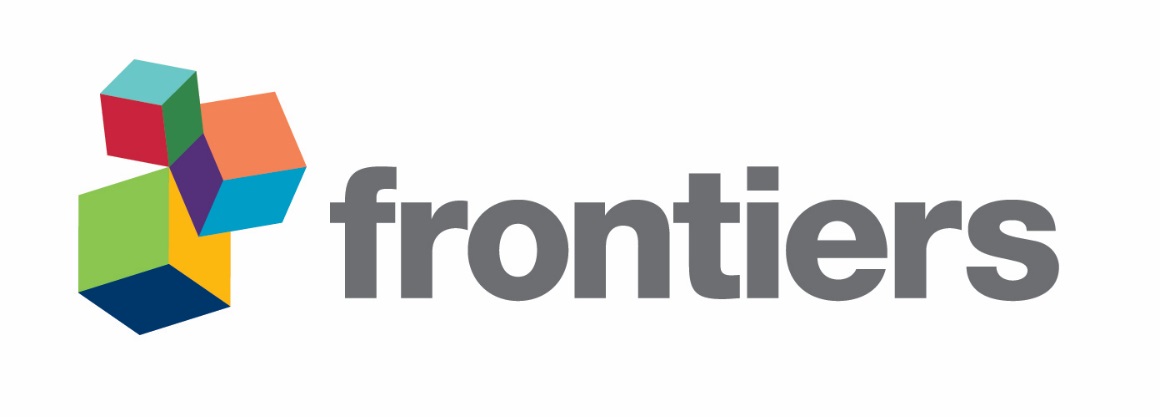
**
